# Matching response to need: What makes social networks fit for providing bereavement support?

**DOI:** 10.1371/journal.pone.0213367

**Published:** 2019-03-07

**Authors:** Samar M. Aoun, Lauren J. Breen, Bruce Rumbold, Kim M. Christian, Anne Same, Julian Abel

**Affiliations:** 1 Palliative Care Unit, School of Psychology and Public Health, La Trobe University, Melbourne, Victoria, Australia; 2 The Perron Institute for Neurological and Translational Science, Perth, Western Australia, Australia; 3 School of Psychology, Faculty of Health Sciences, Curtin University, Perth, Western Australia, Australia; 4 School of Occupational Therapy, Social Work and Speech Pathology, Faculty of Health Sciences, Curtin University, Perth, Western Australia, Australia; 5 Compassionate Communities UK, Honorary Senior Researcher, University of Bradford, Bradford, United Kingdom; University of Indianapolis, UNITED STATES

## Abstract

The objectives of this study were to explore the goodness of fit between the bereaved peoples’ needs and the support offered by their social networks; to ascertain whether this support was experienced as helpful or unhelpful by bereaved people; and to explore both the types of social networks that offer effective support and the characteristics of the communities that encourage and nurture such networks. This study was based on qualitative interviews from twenty bereaved people, in Western Australia, interviewed in 2013. A framework analysis of these interviews was undertaken using a deductive approach based on the goodness of fit framework. Much of this support is provided informally in community settings by a range of people already involved in the everyday lives of those recently bereaved; and that support can be helpful or unhelpful depending on its amount, timing, function and structure. Improving the fit between the bereaved person’s needs and the support offered may thus involve identifying and enhancing the caring capacity of existing networks. An important strategy for achieving this is to train community members in mapping and developing these naturally occurring networks. Some such networks will include relationships of long standing, others may be circles of care formed during a period of caring. Peer support bereavement networks develop from these existing networks and may also recruit new members who were not part of the caring circle. The findings endorse social models of bereavement care that fit within a public health approach rather than relying solely on professional care. As exemplified by Compassionate Communities policies and practices, establishing collaboration between community networks and professional services is vital for effective and sustainable bereavement care.

## Introduction

Bereavement can be an extremely stressful and difficult experience for an individual [1, 2]. Adverse outcomes can occur across multiple domains: emotional (e.g., sadness, anger, guilt), physical (e.g., fatigue, agitation, pain), behavioural (e.g., sleep and appetite disturbance, absentmindedness), and cognitive (e.g., disbelief, confusion, hallucinations). Alongside these consequences, the bereaved must also cope with the secondary losses that arise as a result of bereavement, including social isolation and stigma [3], financial loss [4], and changes of roles and responsibilities [5]. Additionally, bereavement is associated with an increased risk of mortality [6], a higher risk of suicide and suicidal ideation [4] and a higher risk of developing mood, anxiety, and adjustment disorders, and complicated/prolonged grief reactions [7].

Bereaved individuals often seek and receive social support [8], understood here as “an exchange of resources between at least two individuals perceived by the provider or the recipient to be intended to enhance the well-being of the recipient” [9, p.19]. With this definition, social support does come from formal and informal caregivers. Although the receipt of social support is typically viewed as a protective factor in bereavement [10, 11], it is not necessarily helpful and can even lead to the bereaved experiencing more distress [12]. Differentiating beneficial from harmful social support involves more than distinguishing helpful and unhelpful activities, for the same activity that is helpful for one may be regarded as unhelpful by another [13]. The bereaved individual’s perception of helpfulness or unhelpfulness of support is thus important [12, 14]. It is social support that is perceived by the bereaved recipient to be helpful that is important, not the provision of social support itself [1, 14], and unhelpful social support is linked to depression [15]. These ambiguous outcomes of social support underline the need for further exploration of offering and receiving support. Weenolsen’s work [16] reminds us that, as loss may present itself in a number of domains and associated losses, support is needed in all these aspects of the bereaved person’s experience if loss is to be transcended.

A population-based study of bereaved adults demonstrated that the primary sources of bereavement support for most people are their existing social networks, supplemented for some by networks formed during a period spent caring for a dying family member [17]. These additional supporting caring networks may not have formed where an unexpected death has occurred e.g. due to suicide, drowning, accident or homicide. In networks pre-dating dying and bereavement, family and friends were the top sources of bereavement support, followed by financial or legal advisors and religious or spiritual advisors. The community-based networks which formed during the caregiving period typically included the general practitioner, nursing home, hospital, pharmacist, community group, palliative care service, and school-based advisor. Funeral providers were a highly-valued source of support in bereavement. It was not always clear whether these community-based practitioners continued to provide direct support during bereavement, or whether it was the memory of their contribution earlier in the bereavement that continued to sustain the bereaved person. A few people, particularly those in the high risk group of complicated or prolonged grief, sought support during bereavement from professional sources such as a facilitated bereavement support group or a mental health professional such as a social worker, psychologist or psychiatrist [17]. The data from this survey provided empirical evidence for building a community’s capacity to provide the type of social and practical support advocated by the Compassionate Communities approach [18] which relies on identifying and developing local caring networks around the dying person and their family. Compassionate communities normalize seeking and accepting help from family, friends, neighbours, schools and workplaces for emotional and social support not just for end of life support but as part of everyday living [11, 19–21].

As noted above, the social and practical support offered to bereaved individuals may not always meet their needs. In examining the ‘fit’ between the need for and satisfaction with six types of functional social support in relation to bereavement outcomes, Bottomley, Burke and Neimeyer showed that satisfaction with practical support in the months following loss through homicide was the best predictor of favourable bereavement outcomes [1]. With those bereaved by homicide, there are specific impacts on the social network and the grief response (eg anxiety, awkwardness, perception of voyeurism/intrusiveness) which might affect availability/access/satisfaction with social support. It is important, however, to note that Bottomley et al. investigated the functional types of support provided independent of the relationships and contexts in which this support occurred [1]. This neglects evidence suggesting that the perceived effectiveness of support depends on a positive fit between the provider and the recipient of the support such that the same functional type of support may be found by some to be helpful, by others unhelpful. For example, some people find advice giving unhelpful, others find it to be beneficial [22].

Several ‘Goodness of Fit’ factors of social support have been identified as having a role in determining whether social support is perceived by the recipient as helpful. These factors are the source, amount, structure, timing, and function of support.[23] *Sources* of support found in the bereavement survey have already been investigated in previous reports [17, 18, 24]. The *amount* of support has to meet the recipient’s needs; too much or too little can determine whether it is perceived as helpful or unhelpful. The *structure* of social support relates to how connected the members of the recipients’ social networks are and the extent to which they too have been affected by bereavement, which can affect the helpfulness of the support they intend to offer [25]. The helpfulness of social support can also be related to when it was received; some forms of support are more helpful at particular *times* than others. Function relates to types of support, whether informational, instrumental, emotional or appraisal. Although Vachon and Stylianos proposed their model in 1988, there are no studies that have tested this model using empirical data.

### Objectives

The current study is based on qualitative accounts from interviewed bereaved people. Its objectives are:

- to explore the goodness of fit between the support offered by the bereaved social networks and the needs of the bereaved, in terms of the source, amount, timing, structure, and function.
- to ascertain whether the support related to these factors was experienced as helpful or unhelpful by bereaved people.
- to explore the types of social networks that offer effective support and the characteristics of the communities that encourage and nurture such networks.

## Method

### Ethics approval was granted by curtin university research ethics committee (HR 57/2012)

In-depth face to face interviews were conducted with twenty bereaved individuals in Western Australia (WA) in 2013. This qualitative study formed part of a larger study on bereavement support where data were collected through an anonymous postal survey with participants recruited from databases of funeral providers, and who had been bereaved 6–24 months (Aoun et al, 2015). At the end of the survey questionnaire, respondents who wished to take part in an in-depth interview, were invited to leave their name, contact details and the best time to be contacted. There was information within the survey indicating that at most thirty respondents would be invited for an interview. One hundred and ninety-three respondents expressed their interest to be interviewed or 43% of those who completed the survey in WA (n = 447).

Purposeful sampling was used in order to include a variety of characteristics in terms of the setting whether rural (n = 8) or metropolitan (n = 12), the age of the bereaved (40–79 years) and their relationship to the deceased (4 mothers, 2 fathers, 4 daughters, 5 husbands, 5 wives), their bereavement risk (2 low, 13 medium and 5 high as determined from the survey tool on prolonged grief PG-13), whether they perceived receiving enough support from all sources after the death of their relative as determined from the survey (9 had enough support, 11 not enough support) (Table 1). Other variables included the age of the deceased (18–95 years) and gender of the deceased (11 males and 9 females), the cause of their death (eg cancer, heart disease, stroke, accident, suicide, homicide) and whether the death was expected (10 unexpected and 10 expected). (Table 1)

**Table 1:** Characteristics of the bereaved and their deceased relatives.

ID	Bereaved age (yrs)	Relation-ship to deceased	Deceased age (yrs)	Deceased gender	Cause of death	Expected or unexpected death	Bereavement Risk category	Support received
1020M	51	Mother	18	Male	Homicide	unexpected	High	Not enough support
1254M	79	Wife	80	Male	Stroke	unexpected	High	Enough support
1212M	57	Mother	27	Male	Injury/ accident	unexpected	High	Not enough support
1360M	52	Wife	62	Male	Pneumonia following cancer therapy	unexpected	Medium	Not enough support
1075M	63	Wife	63	Male	AML and mesothelioma	expected	Medium	Enough support
1087M	65	Wife	63	Male	Cancer	expected	Medium	Not enough support
1185M	51	Daughter	91	Female	Heart disease	expected	Low	Not enough support
1089M	49	Daughter	75	Male	Lung disease	expected	Low	Enough support
1312M	40	Mother	9	Female	Drowning linked to epilepsy	unexpected	Medium	Enough support
1301M	63	Husband	65	Female	Meningitis/ventriculitis	unexpected	Medium	Enough support
1013M	73	Husband	76	Female	Heart disease	expected	Medium	Not enough support
1444M	67	Husband	62	Female	Secondary breast cancer	expected	Medium	Enough support
1291R	72	Husband	68	Female	Intracerebral haemorrhage into cerebellum	unexpected	Medium	Enough support
1098R	69	Father	40	Female	Leimyosarcoma	expected	Medium	Enough support
1385R	66	Daughter	95	Female	Neurological, bowel cancer, leukaemia	expected	Medium	Not enough support
1127R	47	Father	19	Male	Suicide	expected	Medium	Not enough support
1382R	48	Daughter	80	Male	Cancer	expected	Medium	Enough support
1119R	59	Mother	31	Male	Suicide	unexpected	High	Not enough support
1183R	40	Wife	42	Male	Injury/accident	unexpected	High	Not enough support
1023R	74	Husband	66	Female	Aneurysm	unexpected	Medium	Not enough support

Note: M = metropolitan and R = Rural

The interviewer contacted by phone the selected survey participants who had agreed to participate in the interview phase, provided an explanation about this phase of the study, and set appointments. The information sheet and consent form were posted prior to the interviews. All participants signed their consent forms and handed theirs to the interviewer before the start of the interview. Data were collected face-to-face using a semi structured interview protocol. Interviews were conducted five months from the survey date and the same interviewer conducted all interviews in the home of the interviewees or in an agreed location. The taped interviews were of 23–66 minutes duration (mean 42.00 minutes and SD 12.76). However, the interviewer spent considerable longer times with the interviewees building rapport at the start and then debriefing after the interview (90–180 minutes). The in-depth interview elicited bereaved participants’ thoughts and feelings in relation to their loss, the impact of supports and also the helpfulness or unhelpfulness of their supports prior to and immediately after their relative’s death. Examples of the questions included: “What form of support or help have you received from friends, family, and others? Was this support helpful or unhelpful and in what ways?” The interview protocol is uploaded as a “supporting information” file (S1 File).

## Analysis

We undertook a framework analysis of these interviews using a deductive approach [26, 27] based on the goodness of fit framework [23]. Interviews were digitally recorded, professionally transcribed and de-identified. One co-author conducted the initial coding of the relevant sections of the transcripts which was then verified by another co-author. Emerging themes, patterns, and sentiments were then uncovered and identified to determine the impact of the social supports on the bereaved in relation to the goodness of fit factors (source, amount, timing, structure, and function).

### Findings

The deductive analysis clearly identified the four main factors relating to the goodness of fit framework (beyond the Source): Amount, Timing, Function, and Structure. The analysis also revealed that these factors were interrelated and therefore, some overlapping between the factors existed.

### Amount

The amount of social support received was found to be an important factor in determining whether the participant perceived the support as helpful. For some participants, when there was too much informal support they found it overwhelming and intrusive and hence the support was often perceived to be more of a burden than helpful. This was illustrated by one participant’s comment:

*Trying to be helpful; people were trying to do everything for me and get me involved in this and visit there and that and in the end I just had to say “I’m sorry but I can’t do it”*. *It was easier to come home to a lonely house than it was to cope with another visit to somewhere else for tea that was totally foreign and have to talk about it again or whatever* …(P1075)

Although participants expected support from people they had close relationships with or from people in the community who knew them, several participants did not receive as much support as they would have liked. As an example of this, another participant reported that her brother offered her no support and that consequently she had to deal with everything herself during this stressful time:

*Look it was*, *it was a busy time and I got no help from my brother at all*. *He isn’t helpful and to this day isn’t helpful*, *so I’ve been left with it all*. (P1385)

Yet another participant explained that she felt the community, including her doctor, was very judgemental and offered no support:

*You know*, *my doctor*, *devastated me; my doctor who I’d been with for years and years and years; I wasn’t one to go to the doctor on a regular basis*. *It was almost like I was having a tantrum*. *“Stop your tantrum and just get yourself back to work because it will do you the world…” no sympathy …* (P1020)

In contrast to this, a different participant expressed her satisfaction with the amount of social support she received from informal sources. This participant felt that her, and her partner’s, social support needs were met because their family and friends understood how much support they required:

*I just prefer my closest family with me and the support we had from our friends luckily I think they knew where we stood and they gave us the amount of support we needed*. *If we needed more we could always ask and I think from friends and then our daughter was family and then in [husband name] I had all the support that I needed*. (P1127)

### Timing

Several participants spoke about the timing of the social support they received and how this was helpful. Some participants thought that the ideal time to receive support is not immediately after bereavement but later in the process. Some of the types of support that were helpful at a later timeframe were: Social support from friends, family, work colleagues and the community to alleviate continuing loneliness and isolation and to have the opportunity to talk about the deceased after the initial bereavement; organised support groups; practical help with chores in the home that the partner had previously attended to; social phone calls and visits from friends of the deceased; and being involved in volunteering and helping others. One participant felt that a few months after bereavement is the best time to be offered support because by that time most people are able to talk about their experience:

*I think that is the best way to go and speak to somebody*. *Not necessarily straight away; maybe some people can’t talk about it straight away*, *but I think somewhere in a few months you should go and speak and put it in its place where it needs to be I think*. (P1382)

In contrast, other participants felt that support was helpful when they received it early on and that it became less helpful as time passed:

*At times I think it was good*, *at other times you’re just not interested; you want everybody to just leave you alone*. *I think the first couple of days after [husband’s name] passing it was probably good; maybe the first week or two and then after we didn’t really want to see anybody or talk about it*. (P1127)

Interviewees were asked specifically about their own experience of social support during bereavement. At times, they also reflected on the impact of the death on their immediate family, extended family and friends and their experiences of support, hence the use of ‘we’ in the previous quote.

Another participant illustrated that support should be offered more than once, and that those offering should not assume that refusal at the start means that support will never be needed:

*…but as far as support goes; everyone says “if I can do anything for you”*, *but they don’t offer again. This is what they say in that first couple of weeks, but once the funeral has been no-one comes and knocks on the door “what do you want me to do?” (P1183)*

Other participants again expressed how they valued the ongoing support after bereavement from both informal and community sources. One participant commented on how supportive his church has been, *“*… *I’ve got my church and I’ve got my support there and really tremendous support and they’ve never stopped in two years*” (P1444). Another participant experienced ongoing support from her chemist: *“Chemist*, *the chemist down here at [area name] is amazing; so supportive and he still asks me now how I am and how are you going*, *good days*, *bad days”* (P1360).

### Function

Analysis showed that the social support participants received has served different functions according to whether the support offered was instrumental, emotional, informational, or appraisal support. Participants spoke about the helpfulness of instrumental support provided by informal sources. For example, this participant expressed how beneficial it was having his son take control of the situation and organise what needed to be done:

*My son was fabulous; our oldest son*. *He just went back and got a list of all the; he did have a list for me of all the people I had to contact to let them know what had happened; all the periodical payments*, *tax; he did this big list and he started making the phone calls himself rather than me doing it*. (P1444)

Other participants appreciated instrumental support such as handling funeral and wake arrangements, providing meals, sorting out probate or simply getting their loved ones back on their feet.

*And then after the funeral my sister stayed longer with me and she was fantastic; it’s my eldest sister and she said “let’s get you into a routine. What we’ll do is we’ll make a diary every day” and she said “just simple tasks like get up, have a shower, got to walk the dog every morning*, *have some breakfast then lunch” so just things like that I still found it very hard to eat. (P1360)*

Participants also shared their experiences of receiving emotional support from both informal and community sources. For this participant, knowing that her friends and family were there to offer her emotional support was comforting, *“Oh look*, *they’re just there if you feel like talking or you know*, *they’re just there; they just support you emotionally”* (P1089). Another participant valued emotional support from the community in the form of a card and phone call:

*…the doctors and the paediatrician and her neurologist gave me a personal phone call*. *He was in so much shock because he was on annual leave*. *Between all the specialists in the community*, *they were great*. *The local doctor sent a card*, *so yeah the local community was very good to me*. (P1312)

Informational support provided by informal, community, and professional sources was perceived as helpful by several participants. Some of the forms of helpful informational support were: Targeted brochures and details about support groups specific to the participants circumstance eg a support group for the sudden death of a young child; pamphlets and websites on bereavement and grief reactions; written advice on children attending at funerals; information from schools on managing children’s experience of bereavement; formal information on wills and estates, executors and banking in relation to the death; updates and reports on the circumstances and cause of unexpected deaths and the ongoing legal and/or financial processes.

Some participants found the information provided by funeral providers helpful, *“They were actually the people who had all the pamphlets about the bereavement and who you can contact and all of that*. *That was pretty good”* (P1127). Others spoke about their community providing them with information. One participant found it helpful to be able to speak to someone in the community to get answers without being judged:

*The pastoral care people at [hospital]*. *I knew them because of me being involved down there and they offered great support*. *I could just go and talk to them and try and get explanations and what have you which was excellent*. *It was very helpful to have someone to sit down and talk to; you didn’t have to worry about what you said; just spurt it all out*. (P1291)

Professional sources of support also provided information to the bereaved. However, one participant reported that she had expected counsellors to provide her with problem solving information, but she did not receive this support and so found them to be unhelpful:

*I think I gave counsellors a crack at me because I thought*, *I can’t be stoic all the time and maybe I could see things differently*, *but to me a counsellor is supposed to give you tools and ideas on how to deal with things*. *I never got that in the three people that I saw*. (P1212)

Participants also spoke about receiving appraisal support from both community and professional support sources, often for social comparison purposes. For example, a participant who had lost her mother explained that receiving support from others who had also lost their mothers allowed her to talk freely about her loss:

*Yeah this woman*, *who I’ve met*, *a new friend through a friend; she lost her mother this year so we started talking as you do when you lose your mother and so she said to me “would you like to join our group of Mothers without Mothers*? … *So we go out for lunch on the day of our mother’s birthday*. *Not the day of our mother’s passing* …*then I got to talk all about my mother and then they told me about their mother*. *Well this is a fantastic group of friends because we talk about our mother and it’s ok to sit there for the whole lunch and talk about your dead mum and no one’s going to “oh god can we get off the dead mum subject you know*?*”* (P1185)

Professional sources also provided some participants with appraisal support. One participant found it helpful to speak to a professional about what she was experiencing so she could better understand her grief. However, timing played an important role in determining whether the participant found this support helpful:

*Sometimes it was positive in a sense that it was nice to go somewhere private and talk to someone about how I felt*. *They didn’t judge me*, *they didn’t know me*. *That was capable of; or I could ask a question and I’d say I feel like this and then them sort of explain; this is why you’re feeling like this and this is going to be this and then other times I didn’t like going because I didn’t want to talk about it and it upset me and it put me back a step*. (P1020)

### Structure

The structure of the social support networks of the bereaved was identified as important for determining whether the social support was helpful. Participants spoke about how close their friendship networks were. One participant explained how her close-knit group of friends was able to use their network to offer help. This meant participants did not need to rely only on their family for support:

*I’m just so lucky that I had a really good network of school mums and work that I didn’t really need a family to—and family were great*, *but I just think the combination of it all*, *because we’ve all got to get on with day to day; I just feel very fortunate because the school mums cooked for two weeks and did a roster and I didn’t expect that because there’s no way I was cooking*. (P1312)

Another participant reflected how friends were able to use their close network to offer her effective support:

*So*, *you know*, *my close-knit group of friends were there and they dropped everything*, *left work and rushed to help me and I actually checked myself into the [area name] Mental Health unit and I got in there and they like*, *had organised a room and everything for me and I started to look around*. (P1360)

Some participants reported how their informal support networks were also distressed by the loss, which may have affected their ability to provide helpful support. One participant spoke about how her former husband was not able to support her because he was also grieving, “*My former husband or [the dead person’s] dad; he was sort of; he wasn’t supportive but he was going through the same thing* …*”* (P1212). Another participant explained that he was able to turn to most of his family for support, but not his daughter because she was too distressed. This affected the participant because he felt he had to conceal what he was going through so that he would not upset his daughter:

*If I needed them I could go to them but the real difficult one is my daughter; she was so*, *so very close to her mum*. *They were like sisters rather than mum and daughter and she still gets very*, *very upset and whenever I’m with her I try not to show my own sort of loss because it just sends her off completely; she misses her so much*, *so my relationship with my wife and daughter was so very close*. (P1444)

## Discussion

This qualitative study builds on previous work by our research team in exploring the bereavement experience of a community-based sample and the individual factors that are associated with bereavement [17, 24, 28, 29]. We found that not everyone who receives bereavement support needs it and not everyone who needs bereavement support receives it. It was important to know not only who best provides bereavement support, but more so who is perceived by bereaved people to have offered them the type and amount of support they needed, at the time they needed it. To our knowledge this is the first study to explore all factors of the goodness of fit framework in order to address this question.

Several studies have looked at these factors separately and echoed the experience of the bereaved in this study. Instrumental support such as providing meals and handling funeral arrangements was considered helpful to bereaved participants [3, 12, 30]. Participants in these studies appreciated emotional support which included compassionate gestures such as flowers and cards, behaviours honouring the deceased, and being listened to without any judgement. Informational support can be helpful particularly when sought, but also perceived as unhelpful if insensitive advice is offered [12, 31]. Appraisal support in the form of social comparison as delivered by support groups was considered helpful [8, 12, 32, 33].

The notion that the structure of social support can impact the perceived helpfulness of the support was demonstrated by Dyregrov [34] who found that supporters, who were members of the bereaved parents’ social network, were also dealing with their own grief because they knew the deceased, and that this consequently put strain on the network. Supporters were aware of the possibility of ‘network burn-out’ and some needed to have time-out from supporting the bereaved for self-care purposes [34]. This was also reported recently in the context of family bereavement, with the authors recommending that health professionals/therapists help grievers to focus on creating opportunities to share their grief experiences outside of the family so as to avoid overloading each other’s grief [35].

In the qualitative accounts of this current study, reports of the helpfulness of funeral providers and in some instances the unhelpfulness of counsellors echo some the quantitative findings from the same bereavement survey by Aoun et al [18]. Mental health professionals such as psychiatrists, social workers, case coordinators and school based advisors had the highest proportion of perceived unhelpfulness (33–46%) while about 22% of respondents rated psychologists and counsellors as unhelpful. By contrast, funeral providers, family and friends had the lowest ratings for unhelpfulness (8–12%). It appears that informal social support is most valued as helpful: the emotional bonds (attachment), the practical assistance (tangible alliance) and the perceived sense of belonging (social integration) provided by the existing networks of family and friends are of primary importance [18]. The experiences with funeral service providers reported by the bereaved make it clear that these providers had a vital role to play in bereavement support as one of the community-based assets within the social networks of the bereaved [29, 36]. At a time of major disruption, funeral providers provide a framework for action and undertake a range of tasks that are crucial both to acknowledging the loss and confirming bereaved people in their changed social role.

That some mental health professionals, as reported earlier, had the highest proportion of perceived unhelpfulness deserves further comment. Many of these professionals lack training in loss, grief, and bereavement issues, and often the little knowledge they have is based around the rudimentary 5-stage Kübler-Ross model of emotional adjustment [37]. It is not surprising if bereaved people, finding their counsellors lack a nuanced understanding of their situation, describe some mental health professionals as being unhelpful. This is not to dismiss or diminish the potential contributions of such professionals. Rather, it points to the need for more training and skill development appropriate for working with people experiencing high and chronic distress [38].

In terms of the timing and the amount of social support offered, bereaved people felt sometimes there was too much offering of support which did not meet their needs [39]. Mistimed offers of support may reflect a mismatch between the supporters’ assumptions and a bereaved person’s needs at that time. Stroebe and Schut remind us that bereavement may be described as a dual process, alternating between a focus on the loss and a focus on restoration [40]. Supporters who fail to enquire about or look for indications of where a bereaved person’s attention is focused may address their offers of support inappropriately. Timing is also important in the sense of duration of support. Participants expressed the importance of ongoing support after bereavement because it is common for support to be offered straight after bereavement and then slowly falls away [39]. Both the need to attend to alternation between loss and restoration in the experience of bereavement, and the importance of sustaining care beyond the time leading up to a death, are recognised in a compassionate communities approach to bereavement. Abel and Townsend note that networks of care often disband after someone has died, and they recommend that support be maintained until at least the one-year anniversary of the death [41]. This is even more important given that recent findings from this bereavement survey showed that just half of the bereaved who used palliative care services had a follow-up contact from the palliative care services at three to six weeks, only a quarter had a follow-up at 6 months, and that the standardised routine approach to bereavement support adopted by the services was deemed unhelpful [24]. The need for support networks to connect bereaved people with their lives before loss and with possibilities following loss, not just to focus on the loss itself, also underlines the need to involve bereaved people’s extended networks. Networks formed solely to support the dying process are more likely to focus on the loss of bereavement and have less to offer about restoration. Compassionate communities that engage people over their life span will naturally attend to inclusion, maintenance and restoration: life’s problems will be addressed in the context of life’s possibilities.

Aoun et al have advocated that a public health approach, as exemplified by Compassionate Communities policies and practices [20], should be adopted to support the majority of bereaved people, as much of this support is already provided in informal and other community settings by a range of people already involved in the everyday lives of those recently bereaved [18]. It is important that people need to learn to accept help as refusing it leads to “shutting down valuable support networks at a time when they need to be nourished and built” in end of life care and through to bereavement [42]. As important is the need for the support network to offer help in a more appropriate way, such as being specific with offers of help and making these repeatedly, as exemplified by the quotes. To improve the goodness of fit between the bereaved person’s needs and the support offered, identification of existing networks and enhancement of naturally occurring networks requires training community members in network mapping and development. To this end, citizen volunteering in compassionate communities has been developed, with volunteers recruited, trained and connected in an integrated effort to support people, their families and communities [21, 43, 44].

### Implications for social models of bereavement care

The importance of a population level approach is underlined by Holt-Lunstad et al, who demonstrate that social relationships have a larger impact on reducing mortality than any other existing intervention, over and above giving up smoking, alcohol drinking, exercise, diet, etc [45]. There is a variety of well documented reasons which help explain the impact of compassionate communities. This impact has recently been demonstrated in Frome (UK) where there has been a 30% reduction in hospital admissions as these concepts of community support have been combined with clinical practice [46].

Bereavement arises from the death of a family member or friend, and the majority of these deaths occur in institutional contexts under professional care. The first year of bereavement in particular is marked by an increased number of encounters with professional care providers arising from a range of stress-related health issues [47, 48]. It is thus unsurprising that in recent decades professional care providers have developed an interest in, and a range of responses to, bereavement needs. Nor is it surprising that, lacking effective strategies for referral to informal care networks, professionals can find themselves offering treatment to people whose primary need is social inclusion. Herein lies a dilemma. Offering treatment to those who are struggling with life, whether self-identified or professionally referred, risks obscuring the preventive effect and healing potential of socially inclusive communities. Professional care thus attempts to compensate for support that would ideally be provided through everyday social interactions. However, the lack of socially inclusive communities, and of pathways through which referrals can be made, perpetuates a situation in which need, first identified in health service contexts, is then addressed in those contexts rather than linked back to the informal support of the community. The implication is that care should be shared between professional services and supportive networks according to the expertise of each. Thus, professional services should be aware of, encourage, facilitate and enrich community networks, collaborating with them in mutually understood ways. This has two advantages: The first is that supportive networks are open to everyone while professional bereavement support is available only to a small percentage of those bereaved. Secondly, stretched professional services can reserve their care for those most in need.

It is interesting (although not surprising) that bereaved people often report that they’re not “allowed” to talk about it anymore [3, 49], hence an interview opportunity, like in the current study, where debriefing extended between 90 and 180 minutes, opens the flood gates in providing permission or a space to tell their stories. The high percentage of bereaved respondents to this survey who expressed interest in participating in the interview (almost a half) is likely an indicator of the current dearth of social support they experienced and that, for those people, their networks might not have been as supportive as they could be. Therefore, a public health approach that links personal need with social life and connects formal care with informal support is foundational to a balanced approach to bereavement care.

Our findings endorse social approaches to bereavement care, and we outline here two models of grief that fit in with a public health approach. The first is network enhancement which happens when caring for someone who is dying [50]. Strong, resilient supportive networks can be formed in caring for someone at the end of life, and the number and depth of relationships increases. This enhancement continues to be an important part of social support into bereavement. It is a virtuous circle which resonates for years, maybe even generations [50] and elements of this are indicated in the interview quotes.

The second model is peer support networks into bereavement, such as the Buddy Group in the UK [51] set up to help bereaved people find meaning and value through their grief. Comments in this study described how good it was to talk about the person who had died with other people who had been bereaved, highlighting the need for an approach that does not rely principally on professional care. At the same time, other comments pointed to the problems experienced by some in continuing to mention the bereaved person with family members. While the findings of the Australian survey [18] demonstrated that 41% of bereaved respondents reported that bereavement support groups were of little benefit to them, the mention in these interviews of a peer support group that is specifically for mothers who lost their mothers is an example of how well these groups have to be tailored to specific needs in order to be a good fit. A peer support model can be valuable for people who do not have extensive networks beyond their family, or indeed may not have anyone to talk to. Another key point is that often the people who are most sensitive are the ones who have been through or are going through bereavement themselves. This is probably why peer support groups can be so helpful as they intuitively know what goodness of fit is [49]. More work however is clearly needed in Australia for these support groups to provide a better fit.

Connecting peer support with professional services is a critical factor in improving awareness of and signposting to peer support. The Health Connections Mendip Service Directory [44], which is part of an overall community development approach and has a range of options which can be matched to people’s bereavement needs and preferences, is a good example of how a public health approach can be brought into routine clinical practice [42]. The directory lists assets, including peer support networks, available in the community, and can be consulted during the GP, or other primary care, appointment.

### Limitations

These qualitative accounts come from interviews with twenty people from one geographical area in Australia. Participants were selected on the basis of their willingness to supplement their survey responses, and in order to represent a cross-section of the larger survey population. Their responses however echo the quantitative findings obtained from a national survey of bereaved people [18]. In our discussion we have used the interview data to link with wider explanatory frameworks in the bereavement care and public health palliative care literature and suggest ways in which improvements to bereavement care at a population level might be explored.

The majority of respondents to our survey were people who had experienced an expected bereavement, usually preceded by a period of caregiving [17,24]. While some respondents reported their experience of bereavement following an unexpected death, the number of these was too small to identify a pattern behind the expected individual variations, although it might be noted that sudden or unexpected deaths were more prevalent in the respondents experiencing prolonged grief (64% of unexpected deaths of a total of 6.4% with prolonged grief) [17]. A larger general population sample, or more targeted studies, would be required to find out whether members of bereaved people’s social networks respond differently if bereavement follows sudden death, particularly through accident, homicide or suicide.

Focusing on the small sample of interviewees in this article, the number of those who reported not having had enough support is similar for the two groups of expected and unexpected deaths, though more of the unexpected death group were in the high-risk grief category. This at least suggests that, while there may be differences, we cannot assume that there will be differences in support from social networks just because of the type of death.

## Conclusion

The research literature emphasizes the many negative consequences of bereavement, with increases in physical and psychological morbidity and mortality, and the disruption of social relationships being a primary determinant of both health and mortality. This puts the impact of bereavement squarely into a public health perspective that pursues health equity. We argue here for adopting and strengthening a compassionate communities approach, not only for end of life care for dying people but also along the continuum of bereavement support. To support this approach, more research is needed into practice models that connect health and social services with local neighbourhoods and cultural life. Settings such as workplaces, schools, social and sporting clubs, and faith communities all have potential in making these connections. Fundamentally, however, the issue we have identified here in relation to bereavement is one endemic to contemporary social organisation, a steady increase in social inequality and its negative consequences for well-being [52]. Inequality contributes to social exclusion, particularly for those of reduced means. However, social inclusion is needed not only at end of life, but at all stages of life. Ideally, the networks that support us at end of life will be those that have supported us through life, and the end of life provides both a further opportunity for existing networks to be revived and strengthened, and new networks formed. However, the nature of contemporary society is that those with economic power may retain their social networks and be able to purchase professional support, while those without economic power may lack access to both. As our previous quantitative findings [18] and more recent qualitative findings here indicate, bereavement support is about the circles of care [53] that are formed during the caring process, combined with peer support perhaps from people who were not part of the caring circle. It is imperative that these circles transcend social divisions and become available to all members of the community.

## Supporting information

S1 File Bereavement interview protocol. (DOCX)
