# Supplementary material for: Matching response to need: What makes social networks fit for providing bereavement support?
Source: PLoS One. 2019 Mar 7;14(3):e0213367. doi: 10.1371/journal.pone.0213367 (PMC6405096; doi:10.1371/journal.pone.0213367)
Supplement: S1 File — (DOCX) [file pone.0213367.s001.docx]

**S1 File: Bereavement Interview Protocol**

*I’m here to find out about your experiences from your point of view about losing your loved one. I hope that this information will assist in developing effective supports for people who are bereaved. I would like to spend our time together talking about your thoughts and feelings in relation to your loss, life at the moment, and to discuss the supports you’ve received during this time.*

1. Can you talk about finding out about (your loved one’s) death?

2. Can you describe the days that immediately followed (your loved one’s) death.

(Tell me something about your experience since the death of your loved one?)

3. Can we now talk about Issues or problems you faced in the first few weeks or months after the death (including individual, environmental and situational factors).

Description.

Problem solving.

Examples

4. Can you describe any current issues - issues that you may have *now (including individual, environmental and situational factors)?*

5. How have issues or problems that you have had changed over time?

The following questions are about help or support you have received from friends and family and others but NOT services or support services*. Include help/support for individual, situational and environmental factors).*

6. What form of support/help have you received from friends, family and others?

7. Who helped/supported and how. Examples .

8. Was this support helpful or unhelpful? Explain.

9. Did you find receiving the help/support a negative/positive experience? Were you satisfied with the help/support? Have any new relationships or support taken place during your bereavement?

10. What help/support would have been helpful? Explain.

11. Has your access to help/support been affected by where you live (include rural/metropolitan, distance from family or friends/ no transport etc)?

*12.* Can you describe the reactions of family/friends around you to your loss?

13. What are some of the helpful or unhelpful things they did?

14. What was your reaction to their reactions?

15. How would you have liked them to react?

16. Is there anything else that could have helped?

*Employment/work.*

17. What was the reaction of your and your loved ones boss/colleagues?

18. What were some of the helpful or unhelpful things they did?

19. Can you describe what you actually wanted from them?

20. Is there anything else that could have helped?

*Reactions from the community.*

1. 21. What was the reaction from your community? Where/who from.
2. 22. What were some of the helpful /unhelpful things they did?
3. Examples.

*Support services.*

1. 23. Are you aware of any support services for bereavement that you can access?

*24. Did you access any services? If you did not access them, why not?*

1. *How confident are you that you would call on a service if you felt you needed it? Explain.*
2. *24. What were the support services that you accessed?*
3. 24. Were they helpful or unhelpful? Examples. What else could they have done?
4. 25. Did you find having the support service(s) overall a positive or negative experience? Explain.
5. 26. Do you feel that your access to support services has been affected by where you live (include rural/metropolitan, distance from services, no transport etc)?

27. What do you think would be the ideal support after bereavement?

*28.* Do you have any advice for the bereaved?

29. Are there any positive things that come out of experiencing hard times?

30. Have you experienced any growth and/or changes since you lost your loved one?

31. How do you see your future without your loved one?

*Are there other questions you wished I had asked you?*

*We’ve come to the end of my questions. Thank you for your time.*

How are you feeling? Do you have support that you can access now if you feel you would like support? I have some information and pamphlets of people who are able to talk further with you about any feelings that may have arisen. Follow up with a phone call the following day if the participant is distressed.
